# Safety of measles-containing vaccines in post-marketing surveillance in Anhui, China

**DOI:** 10.1371/journal.pone.0172108

**Published:** 2017-02-13

**Authors:** Fan-Ya Meng, Yong Sun, Yong-Gang Shen, Hai-Feng Pan, Ji-Hai Tang, Bin-Bing Wang, Chang-Hao Wu, Dong-Qing Ye

**Affiliations:** 1 Department of Epidemiology and Biostatistics, School of Public Health, Anhui Medical University, Hefei, Anhui, China; 2 The Key Laboratory of Major Autoimmune Diseases, Anhui Province, Hefei, Anhui, China; 3 Department of Immunization and Prevention, Anhui Center for Disease Control and Prevention, Hefei, Anhui, China; 4 Faculty of Health and Medical Sciences, University of Surrey, Guildford, United Kingdom; Public Health England, UNITED KINGDOM

## Abstract

The safety of measles vaccination is of great interest and importance to public health practice and the general society. We have analyzed the adverse events following immunization (AEFIs) of currently used measles-containing vaccines (including live attenuated measles vaccine, live attenuated measles and rubella combined vaccine, live attenuated measles and mumps combined vaccine, live attenuated Measles, Mumps and Rubella Combined Vaccine) in Anhui Province, China. From 2009 to 2014, 9.9 million doses of measles-containing vaccines were administrated and 1893 AEFIs were found (191.4 per million doses), of which, 33 serious AEFIs (3.3 per million vaccine doses) were reported. 59.4% (1124 cases) were male cases, and 85.1% (1611 cases) occurred in persons aged < 1 year. 93.3% (1766 cases) occurred at the first dose of vaccination and 95.9% (1815 cases) were found within 3 days after vaccination. This study presents up-to-date data and suggests that the measles-containing vaccines used in Anhui Province of China are safe.

## Introduction

Measles is one of the most contagious diseases caused by the measles virus, a paramyxovirus of the genus Morbillivirus[1]. About twenty million measles cases occur every year, and in most countries of the world, measles is endemic[2]. Currently, no specific antiviral treatment is available for measles virus. The most effective way to control measles is through vaccination. Great progress has been made in global control of measles. The estimated number of deaths declined by 74% from 535,300 in 2000 to 139,300 in 2010[3]. However, measles remains one of the leading causes of death among young children worldwide and the current vaccination coverage of 85%, although reaching a historical height, is still below the desired World Health Organization (WHO) target of 95% (WHO: Measles Fact Sheet, March 2016), including some Western countries (Health & Social Care Information Centre: NHS Immunisation Statistics, England 2013–2014; 25th Sept 2014). Therefore it remains a long-term goal to communicate and engage to build public confidence and promote immunization. To this end, evaluation and monitoring of the vaccination safety is an important aspect of the global collaboration towards prevention and control of measles.

China is the largest country in WHO’s Western Pacific Region. Although China has made great efforts in recent years to eliminate measles, the virus continues to spread and cause serious morbidity [4, 5], occurring in most provinces of China [6–9]. The average annual reported incidence of measles in China has decreased from 572.0 per 100,000 from 1960–1969 to less than 10 cases after the year 2000[10]. The average annual reported incidence of measles in Anhui Province has decreased from 13.2 per 100,000 from 1986–1995 to 3.2 cases from 2009–2014[11].

Key to the effective implementation of MCV vaccination is the monitoring of the associated adverse events following immunization (AEFIs) of measles-containing vaccines (MCVs) (including measles vaccine, measles-mumps-rubella vaccine, measles-rubella vaccine, measles-mumps vaccine). These common AEFIs included fever, rash, injection site reaction, seizure, encephalomyelitis, thrombocytopenia, anaphylaxis, acute arthralgia (adults), acute arthritis (adults), parotid swelling and aseptic meningitis in pre-licensure studies and post-marketing studies [12–17]. To maintain effective vaccine safety monitoring of MCV, updated data should be provided in a timely manner, to ensure these vaccines are safe which will help in maintaining public trust in the vaccination program.

MCVs have been widely used in Anhui. However, to date, the AEFIs have not been systematically analyzed. In this study, AEFIs of MCVs (produced by Beijing and Shanghai Institutes) from January 1, 2009 through December 31, 2014 will be described and analyzed in detail, on the basis of reports from China Immunization Safety Surveillance System.

## Materials and methods

### China Immunization Safety Surveillance System

Anhui started to use China Immunization Safety Surveillance System in 2008(http://219.141.175.204/) [18]; this was a passive surveillance system. Vaccinations and AEFIs reports were the important tasks of the Centers for Disease Control and Prevention (CDC) in China and were coordinated centrally. CDCs were distributed throughout the country at different levels–county, prefecture and province with increasing administrative powers. Any events associated with vaccination were reported to CDC, investigated and diagnosed by a group of appointed experts (see “Reporting and investigation of AEFI” for more details). Events categorizing and casualty assignment followed the national guidelines and were standardized with an inspection and quality control system. The data were reviewed monthly, quarterly and annually. The feedback, conclusions and recommendations were made and disseminated to all the organizations and industry involved in the vaccination program.

In Anhui, the system covered all levels of CDC, including 16 cities and 105 counties, and as of 2014, 19983 AEFIs had been reported from this system in Anhui. National guideline for the surveillance of AEFI[19] was issued by National Health and Family Planning Commission of the People’s Republic of China (NHFPC) and China Food and Drug Administration (CFDA) in June 2010, which described how to report AEFIs, what to report and when to report.

In the National guideline for the surveillance of AEFI, based on World Health Organization (WHO) guidelines, an AEFI was defined as the occurrence of events deemed to be caused by the vaccine. In our study, serious AEFIs, on the basis of WHO guidelines, referred to hospitalization, death, life-threatening illness, and permanent disability[12].

### The scope and time limit of reporting

On the basis of National guideline for the surveillance of AEFI, the scope and time limit of AEFIs reporting were divided into the following categories[19]①within 24h: allergic shock, anaphylactic reaction without shock (urticaria, maculopapule, laryngeal edema, etc.), toxic shock syndrome, hysteria, syncope; ②within 5 days: fever (axillary temperature≥38.5℃), redness and swelling in the vaccination site (diameter > 2.5 cm), induration (diameter > 2.5 cm), angioedema, the whole body suppurative infection (pyemia, toxicema, blood poisoning), local suppurative infection (phlegmon, lymphangitis and lymph node phlogistic, local abscess), etc.; ③within 15 days: measles scarlet fever-like rash, febrile convulsion, seizure, encephalitis and meningitis, anaphylactoid purpura, arthus reaction, polyneuritis, etc.; ④within 6 weeks: thrombocytopenic purpura, vaccine-related paralytic polio, Guillain-Barré syndrome, etc.; ⑤within 3 months: sterile abscess in the vaccination site, brachial plexus neuritis, etc.; ⑥others: other serious AEFIs related to the vaccination and any medical events believed to be connected with vaccination. However, if an event was beyond the above time limit, clinicians would report to China Immunization Safety Surveillance System if it was deemed to be caused by the vaccination, and the classification of the AEFIs case was based on his/her diagnosis.

In this study, classification of AEFIs was based on Definitions Criteria of the Brighton Collaboration. AEFIs included Fever(≥38℃, classified as Level 1 of diagnostic certainty), Rash(Level 1 of diagnostic certainty), A local reaction at or near injection site(Level 1 of diagnostic certainty), Anaphylaxis(One case in Level 3 of diagnostic certainty, the remaining cases in Level 1.), Seizure (Level 1 of diagnostic certainty), Diarrhea(Level 1 of diagnostic certainty), Fatigue(Level 1 of diagnostic certainty), Encephalitis/Myelitis(Level 1 of diagnostic certainty), Thrombocytopenia(Level 1 of diagnostic certainty) reported in China Immunization Safety Surveillance System in Anhui from 2009 to 2014[20–28]. Of these, serious AEFIs referred to Anaphylaxis, Seizure, Encephalitis/Myelitis and Thrombocytopenia.

### Reporting and investigation of AEFI

**Fig 1:**
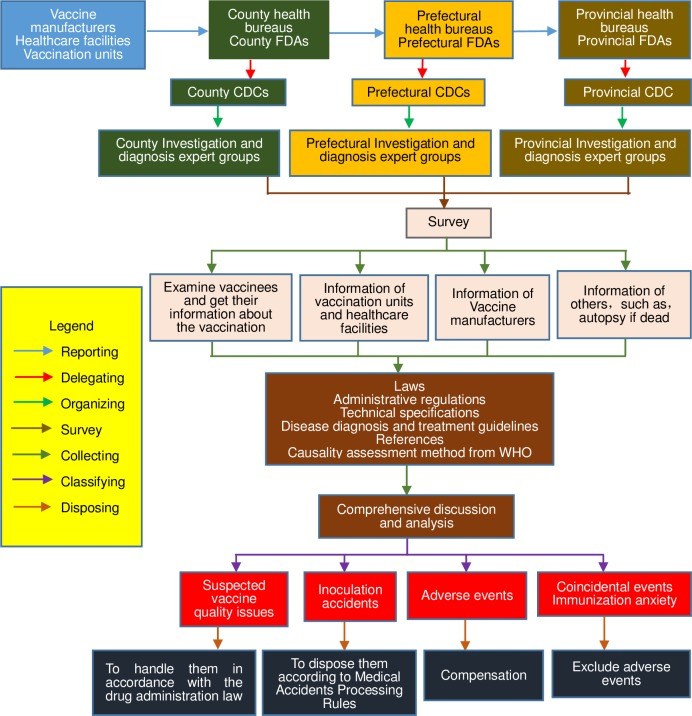
A flow diagram for process for categorising events and assigning causality of adverse events following immunization in Anhui of China.

Medical Institutions, immunization units, CDCs, agencies for monitoring adverse events of drugs, vaccine manufacturers and vaccine wholesale businesses were the responsible reporting units and reporters for AEFIs. When receiving a report of an AEFI, the reporting unit was demanded to fill in a case-report table and present it to the county CDC. After verification, the county CDC should report it through China Immunization Safety Surveillance System. The county CDC staffs should investigate AEFIs and fill in detailed investigation forms for all AEFIs except those with clear diagnosis (e.g. fever, a local reaction). On the basis of the National guideline for the surveillance of AEFI [19], every county-level, prefecture-level, and province-level CDC must establish an expert database to investigate and diagnose AEFIs. When an AEFI occurred, health administrative department should select appropriate experts from the expert database according to the type of the AEFI to form an investigation and diagnosis group. Each group should be composed of epidemiologists, clinicians, pharmacists, and other relevant experts. Causality assessment standards consulted Causality Assessment of An Adverse Event Following Immunization published by WHO, it contained 4 steps: Step one. Eligibility: The first step was to decide if the AEFI met the minimum criteria for causality assessment as shown below. Step two. Checklist: The second step was a systematically reviewing of the available relevant evidence to determine possible causal sides of the AEFI (http://www.who.int/vaccine_safety/publications/aevi_manual.pdf). Step three. Algorithm: The third step followed the WHO algorithm to obtain a trend about the causality with the information collected in the checklist. Step four. Classification: The final step classified the AEFI’s in terms of their association with the vaccination/vaccine according to the trend determined in the algorithm[12]. Usually, prefectural or provincial level experts were responsible for investigating deaths, life-threatening illnesses, and permanent disabilities; county-level experts were responsible for other AEFIs. Information from case-report form and detailed investigation form was reported to the Anhui CDC through China Immunization Safety Surveillance System. All serious AEFIs were investigated by experts of the county, prefectural, or provincial CDCs immediately after receipt of reports (Fig 1).

### Vaccine and data on vaccine administration

The vaccines were derived by using measles virus Shanghai-191, mumps virus S79 attenuated strain, and rubella virus BRDⅡattenuated strain [29–32].

In Anhui Province from 2009 to 2014, MR and MMR were used in routine immunization and MV and MM was used in supplementary immunization.

Data on the number of vaccine administered was reported by township vaccination units to the county CDCs every month; thereafter county CDCs reported the data to prefectural CDCs and prefectural CDCs then reported to Anhui Provincial CDC. We used this data as denominator when calculating the incidence of AEFIs.

### Data analysis

We calculated the incidence of AEFIs as per million doses, and examined the age and gender distribution of the AEFIs. We analyzed dose of the vaccination, time intervals from vaccination to onset of symptoms, simultaneous vaccination, reported symptoms and case diagnosis. If more than one symptom was reported on one person, only the most serious diagnosis or the main symptom was recorded in China Immunization Safety Surveillance System. Serious AEFIs were described in detail for their importance.

## Results

### AEFI after MCV vaccination

**Fig 2:**
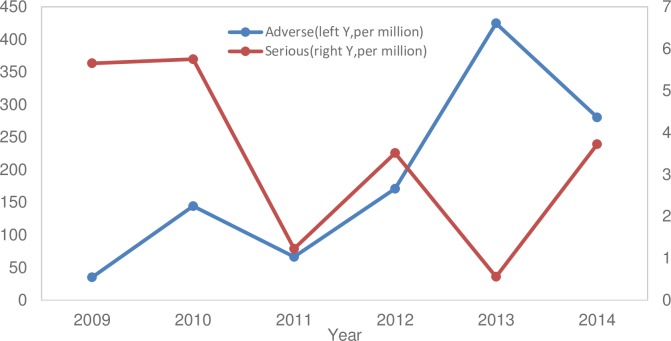
The reported rates of adverse events after immunization with measles containing vaccines in Anhui, China, 2009–2014.

Between 2009 and 2014, a total of 9.9 million doses of the MCVs were used and 1893 AEFIs were reported in Anhui Province. No deaths were reported. The overall incidence rate of reported AEFIs was 191.4 cases per million doses with the annual incidence rate of AEFI ranging from 35.2 per million doses in 2009 to 280.4 per million doses in 2014, with 424.6 per million doses in 2013, which was the highest rate during the study period. There were 33 serious AEFIs; the overall rate was 3.3 per million vaccine doses, with the annual rate ranging from 5.7 per million doses in 2009 to 3.7 per million doses in 2014, with the peak rate of 5.8 per million doses in 2010. The incidence of AEFIs showed a rising trend; however the serious AEFIs presented a downward trend (Fig 2).

### Types of AEFIs

Most of the AEFIs were fever, accounting for 55.8% (106.9 per million doses), rash (48.3 per million doses) and local reactions (29.3 per million doses). There were more male than female cases with a gender ratio of 1.46:1.00, a total of 1124 male cases had an AEFI (59.4% of the total cases). 85.1% of the AEFIs were reported in 8–12 months age group with a total of 1611 cases (Table 1).

**Table 1:** The adverse events following measles containing vaccines (MCVs) in Anhui Province 2009–2014.

AEFIs	No.of cases(per million)	Gender		Age groups				Vaccines received			
		Male	Female	8∼12 months	2∼4years	5∼7years	≥8 years	MR	MMR	MV	MM
		No.of cases(Percentage)									
Fever											
37.0-<37.5°C	137(13.9)	70(51.1)	67(48.9)	121(88.3)	10(7.3)	2(1.5)	4(2.9)	85(62.0)	40(29.2)	12(8.8)	0(0.0)
37.5-<38.5°°C	481(48.6)	306(63.6)	175(36.4)	432(89.8)	35(7.3)	9(1.9)	5(1.0)	318(66.1)	115(23.9)	47(9.8)	1(0.2)
≥38.5°C	439(44.4)	247(56.3)	192(43.7)	369(84.1)	63(14.4)	3(0.7)	4(0.8)	234(53.3)	123(28.0)	80(18.2)	2(0.5)
Local reaction(diameter)											
<2.5cm	153(15.5)	94(61.4)	59(38.6)	127(83.0)	20(13.1)	4(2.6)	2(1.3)	70(45.8)	67(43.7)	16(10.5)	0(0.0)
2.5-<5.0cm	113(11.4)	63(55.7)	50(44.3)	97(85.8)	7(6.2)	2(1.8)	7(6.2)	35(31.0)	73(64.6)	4(3.5)	1(0.9)
≥5.0cm	24(2.4)	19(79.2)	5(20.8)	16(66.6)	7(29.2)	0(0.0)	1(4.2)	5(20.8)	18(75.0)	1(4.2)	0(0.0)
Rash	478(48.3)	289(60.5)	189(39.5)	413(86.4)	46(9.6)	6(1.3)	13(2.7)	317(66.3)	88(18.4)	72(15.1)	1(0.2)
Anaphylaxis	14(1.4)	8(57.1)	6(42.9)	6(42.8)	0(0.0)	4(28.6)	4(28.6)	3(21.4)	3(21.4)	7(50.0)	1(7.2)
Seizure	14(1.4)	6(42.9)	8(57.1)	8(57.2)	4(28.6)	1(7.1)	1(7.1)	4(28.6)	3(21.4)	7(50.0)	0(0.0)
Diarrhea	12(1.2)	8(66.7)	4(33.3)	9(75.0)	1(8.3)	0(0.0)	2(16.7)	7(58.3)	2(16.7)	3(25.0)	0(0.0)
Fatigue	4(0.4)	1(25.0)	3(75.0)	0(0.0)	0(0.0)	0(0.0)	4(100.0)	0(0.0)	2(50.0)	2(50.0)	0(0.0)
Encephalitis/Myelitis	3(0.3)	1(33.3)	2(66.7)	2(66.7)	1(33.3)	0(0.0)	0(0.0)	0(0.0)	1(33.3)	2(66.7)	0(0.0)
Thrombocytopenia	2(0.2)	2(100.0)	0(0.0)	1(50.0)	1(50.0)	0(0.0)	0(0.0)	1(50.0)	0(0.0)	1(50.0)	0(0.0)
others	19(1.9)	10(52.6)	9(47.4)	10(52.6)	4(21.1)	2(10.5)	3(15.8)	5(26.3)	3(15.8)	11(57.9)	0(0.0)
Total	1893(191.4)	1124(59.4)	769(40.6)	1611(85.1)	199(10.5)	33(1.8)	50(2.6)	1084(57.3)	538(28.4)	265(14.0)	6(0.3)

Note: AEFIs—adverse events following immunization.

MV—measles vaccine.

MMR—measles-mumps-rubella vaccine.

MR—measles-rubella vaccine.

MM—measles-mumps vaccine.

As for the incidence of AEFIs of different types of vaccines, AEFIs of MR were 273.3 per million doses, MMR were 137.1 per million doses, MV were 213.9 per million doses and MM were 7.9 per million doses from 2009 through 2014.

### Vaccination doses, reaction intervals and simultaneous vaccination

Majority of the AEFIs (1766, 93.3%) occurred following the first dose of vaccination. A much smaller number of AEFIs were reported following subsequent doses of MCVs: 63 (3.3%) AEFIs after the second dose, 38 (2.0%) AEFIs after the third dose, and 26 (1.4%) AEFIs followed the fourth dose.

The AEFIs commonly occurred within 3 days after MCV vaccination (1815 cases, 95.9%). Overall, 1177(62.2%) cases occurred on the day of vaccination, 638 (33.7%) cases happened between 1 and 3 days after vaccination, 47 (2.5%) cases took place in 4 to 7 days and 31 (1.6%) cases were in 8 to 36 days.

MCVs were administered alone in 1091 (57.6%) out of the total 1893 AEFIs, in combination with other vaccine in 796 (42.1%) events and in combination with two other vaccines in 6 (0.3%) cases. In 802 cases of simultaneous administration, 581 (72.4%) cases followed the first dose of MCVs, and 221 (27.6%) cases followed the second dose.

### Serious AEFI

The 33 serious AEFIs included Anaphylaxis, Seizure, Encephalitis/Myelitis and Thrombocytopenia. A total of 17 male vaccine recipients had an AEFI (51.5% of the total cases). There were 17 cases of AEFIs aged from 8 to 12 months (51.5%), and 6 cases in 2 to 4 years old (18.2%); 5 (15.2%) cases of AEFIs were reported for both 5–7 year group and 8 year plus group.

The top three vaccines associated with serious AEFIs were MV, MR and MMR. MV was associated with nearly half of the serious AEFIs with 17 cases (51.5%; rate 13.7 per million doses), followed by MR with 8 cases (24.2%; rate 2.0 per million doses), and then by MMR with 7 cases (21.2%; rate 1.8 per million doses). There was only 1 case inoculated with MM (Table 2).

**Table 2:** Serious AEFI in Anhui from 2009 through 2014.

Serious AEFI	Total	MR	MMR	MV	MM
	No.of cases(per million)				
Anaphylaxis	14(1.4)	3(0.8)	3(0.8)	7(5.7)	1(1.3)
Seizure	14(1.4)	4(1.0)	3(0.8)	7(5.7)	0(0.0)
Encephalitis/Myelitis	3(0.3)	0(0.0)	1(0.3)	2(1.6)	0(0.0)
Thrombocytopenia	2(0.2)	1(0.3)	0(0.0)	1(0.8)	0(0.0)
Total	33(3.3)	8(2.0)	7(1.8)	17(13.7)	1(1.3)

69.7% of the serious AEFIs were vaccinated with MCVs only, 90.9% of them occurred within 3 days, and also 90.9% followed the first dose. The seizure cases were short lived, generalised tonic-clonic in nature.

## Discussion

This report provides updated 6 year data on the AEFIs of MCVs in Anhui Province from 2009 to 2014 and suggests MCVs have a very good safety profile. The rate of reported AEFIs after measles-containing vaccines from 2009 through 2014 in Anhui was 191.4 per million doses which was below the national rate of 338.1 per million doses in China (calculated from 2010–2014 data, [33–38]), and there were no deaths. The specific rates of AEFIs for MR, MMR, MV and MM were also lower than the national rates [33–38]. The rate of serious AEFI cases was 3.3 per million vaccine doses, similar to 3.7 per million vaccine doses for the whole China [33–38]. This is an area that needs further study in future monitoring efforts. It is of interest to note that data during this period of MCV program did not show a pattern or cluster of AEFIs that would be concerning. Further more, most of the AEFIs were minor reactions (e.g. injection site reactions, fever) which have been observed in the pre-licensure studies and other post-licensure studies [13, 15–17]. The demonstrated safety profile of the MCV vaccines from our study is re-assuring to public health providers and the general public. As the same sources of MCV vaccines have been used many other provinces in China, this data may also serve as a reference base for MCV program in other regions of the country.

Both individual categories of common and serious AEFIs in our report also had lower rates than the international background data. The top three AEFIs reported in Anhui Province from 2009 through 2014 were Fever (106.9 per million doses), Rash (48.3 per million doses), Local reaction (29.3 per million doses). These were much lower than the corresponding incidences mentioned in Immunization Safety Surveillance, which were 5–10% for Fever, 5% for Rash and 17–30% for Local reaction [12]. Similar trend was also seen in the serious AEFIs. The incidences of the serious AEFIs (Anaphylaxis, Seizure, Encephalitis/Myelitis and Thrombocytopenia) in our study were 1.4, 1.4, 0.3 and 0.2 per million doses respectively. These were lower than the corresponding incidences in Immunization Safety Surveillance which were 1–3.5 per million doses, 1 in 2000–3000 doses, 1 per million doses and 1 per 30000 doses respectively [12].

Of note, 59.4% of the AEFI cases were males. It could be a real biological phenomenon but could also be partly due to biased reporting. A majority of the AEFIs were reported in the children between 8–12 months old and also happened after the first dose of MCVs. In China, routine immunization schedules require that the first dose is inoculated when children grow up to 8 month old, and the second dose is administered when they reach 18–24 months. This data suggests that the first dose of MCVs may have higher risk of AEFIs than the second dose. However, the higher risk of AEFIs in the first dose may be partly due to the vaccination form, as the first dose of MCVs had a greater percentage (72.4%) of combined vaccines than the second dose (27.6%). This was also seen in other studies [16, 33]. Irrespective of the precise reasons, the message here is that the surveillance of AEFIs of MCVs should pay more attention to children under 1 year old and also to the first dose.

Approximately 95.9% of AEFI cases happened within three days after the vaccination. This could be partly due to the fact that reactions that happened within a short time after the vaccination were more likely to attract attention from children's guardians and the doctor. On the other hand, the report deadline requirements (e.g. only local reaction and fever within five days after vaccination were demanded to be reported as AEFI) may also play a role. Similar observation was also made in other studies [16, 33–38].

Improvement to the surveillance system following introduction of the National surveillance program in 2010 and the detailed Anhui provincial guidelines in 2013 would have only improved the detection rate of the AEFIs and the accuracy of these AEFIs, especially the serious events. However, these changes to the surveillance system did not seem to have a significant effect on the rates of overall AEFIs and the serious events. Specifically, a small rise in the rate of total AEFIs in 2010 was followed by a return of the rate in 2011 which was similar to that in 2009; a dip in the rate of the serious cases in 2013 was again followed by a recovery of the rate in 2014 to a level similar to that in 2012.

Some limitations in this study must be acknowledged. China Immunization Safety Surveillance System was passive so that it was possible that some AEFIs might not have been reported by this system; therefore there could be under-reporting. Furthermore, most of the AEFI cases were found within 3 days following the vaccination, suggesting that there could be a bias when reporting AEFI cases, an inherent limitation of a passive reporting system. In addition, this study was a descriptive research and hence limited our ability to ascertain a causal relationship between the AEFI and the vaccine. Nevertheless, the overall rate and the incidence of specific types of AEFIs were well within the range reported elsewhere.

To sum up, we did not observe a pattern or cluster of AEFIs that may be concerning. These results demonstrate that MCVs administrated in Anhui had a very good safety profile.
